# Experiences and Views of Young People and Health Care Professionals of Using Social Media to Self-Manage Type 1 Diabetes Mellitus: Thematic Synthesis of Qualitative Studies

**DOI:** 10.2196/56919

**Published:** 2024-05-29

**Authors:** Yanan Ma, Kate Law, Lamiece Hassan, Goran Nenadic, Sabine N van der Veer

**Affiliations:** 1 School of Computer Science The University of Manchester Manchester United Kingdom; 2 Division of Nursing, Midwifery & Social Work The University of Manchester Manchester United Kingdom; 3 Division of Informatics, Imaging and Data Sciences The University of Manchester Manchester United Kingdom

**Keywords:** adolescents, health care professionals, social media, thematic synthesis, type 1 diabetes, type 1 diabetes mellitus, T1DM, young people

## Abstract

**Background:**

Social media have shown the potential to support type 1 diabetes self-management by providing informational, emotional, and peer-to-peer support. However, the perceptions of young people and health care professionals’ (HCPs) toward the use of social media for type 1 diabetes self-management have not been systematically reviewed.

**Objective:**

The aim of this study is to explore and summarize the experiences and views of young people with type 1 diabetes and their HCPs on using social media for self-management across qualitative findings.

**Methods:**

We searched MEDLINE, Embase, PsycINFO, and CINAHL from 2012 to 2023 using Medical Subject Heading terms and text words related to type 1 diabetes and social media. We screened and selected the studies according to the inclusion and exclusion criteria. We quality appraised and characterized the included studies and conducted a thematic synthesis.

**Results:**

We included 11 studies in our synthesis. A total of 9 of them were qualitative and 2 were mixed methods studies. Ten focused on young people with type 1 diabetes and 1 on HCPs. All used content analysis and were of moderate to high quality. Thirteen descriptive themes were yielded by our thematic synthesis, contributing to five analytic themes: (1) differences in how young people interact with social media, (2) characteristics of social media platforms that influence their use and uptake for type 1 diabetes self-management, (3) social media as a source of information, (4) impact on young people’s coping and emotional well-being, and (5) impact on support from and relationships with HCPs and services.

**Conclusions:**

The synthesis suggests that we should consider leveraging social media’s peer support capabilities to augment the traditional services for young people with type 1 diabetes. However, the patients may have privacy concerns about HCPs’ involvement in their online activities. This warrants an update of existing guidelines to help young people use social media safely for self-managing their diabetes.

## Introduction

Type 1 diabetes mellitus (T1DM) is the most common form of diabetes diagnosed in children and young adults, with a global prevalence of 5.9 per 10,000 people per year [1,2]. Children with T1DM have a 12-year reduced average life expectancy, mainly as a result of acute and chronic complications (eg, hypoglycemia, as well as cardiovascular and cerebrovascular diseases) [3-5].

Reducing the incidence of complications and improving diabetes-related quality of life relies on effective self-management [6,7]. This includes psychosocial acceptance of living with T1DM and tasks such as home blood glucose monitoring and adhering to complicated medical regimens [8]. Yet, many young people struggle to self-manage effectively and find it overwhelming [9], while also often enduring depression, anxiety, stigma, discrimination, and inadequate support [10-13]. Moreover, traditional health care services may not fully meet their needs because of long waiting hours, scheduling problems, and a fear of being judged [14].

Social media platforms have shown potential to support self-management for several long-term conditions, including T1DM [15-19]. For example, Meade et al [20] suggested that social media could help build diabetes-related skills and knowledge by providing patients and health care professionals (HCPs) access to information and means to interact with each other. In addition, social media platforms can be a source of emotional support [14] by facilitating contact with peers for validation and sympathy, which can increase their sense of normality and belonging [21,22]. Finally, the anonymity offered by social media (eg, use of pseudonyms) can enable young people to discuss sensitive topics with less fear of embarrassment or judgment [23-25].

At the same time, however, information shared on social media can be inaccurate, inappropriate, or misrepresented [8,26], and there are concerns about privacy and security when sharing personal health information online [27,28]. Given the presence of both potential benefits and risks, it is important to further understand young people’s and HCPs’ experiences, views, and concerns about using social media to self-manage T1DM. Previous qualitative studies explored this [14,25,28] but were limited to a particular time, geography, demographic group, and context [29]. A review and summary of findings across qualitative studies is still lacking.

We, therefore, aimed to address this gap by (1) identifying and characterizing the studies that examined young people’s and HCPs’ experiences and views of using social media to self-manage T1DM and (2) exploring and synthesizing these experiences and views, as well as the reasons for young people to use or not to use, or for HCPs to recommend or to not recommend social media for self-managing T1DM.

## Methods

### Thematic Synthesis

Thematic synthesis is a way of integrating findings from multiple qualitative studies and providing a wide range of perceptions of people in different time, space, population groups, and contexts [30] by synthesizing quotations and findings from each study. We used a thematic synthesis approach for summarizing young people’s and HCPs’ experiences and perceptions of using social media to self-manage T1DM as reported in previous qualitative studies. With this, we aimed to produce a new, higher-level understanding of this phenomenon beyond the original findings of individual studies.

The design of this review was informed by Thomas and Harden’s [29] guidelines and reported in line with the PRISMA (Preferred Reporting Items for Systematic Reviews and Meta-Analyses) guidelines [31], where relevant. We registered this review on PROSPERO [32], which is an international prospective register platform for systematic reviews.

### Identifying Relevant Studies

#### Search Strategy

We searched MEDLINE, Embase, and PsycINFO via Ovid, as well as CINAHL on February 16, 2023, using Medical Subject Heading terms and text words related to T1DM and social media (see Multimedia Appendix 1). We selected search terms based on previous reviews on related topics (eg, Elnaggar et al [8] and Faulds et al [26]) and refined them with the support of a librarian. The search was restricted to studies published in English since 2012, which was when social media became more mainstream. To complement our electronic search strategy, we manually searched reference lists of included studies.

#### Inclusion and Exclusion Criteria

Our criteria for selecting papers, structured according to population, phenomenon of interest, and context [33] are mentioned in Table 1.

**Table 1:** Inclusion and exclusion criteria.

Inclusion criteria		
	**Population**	
		People aged 10-24 years diagnosed with T1DM^a^
		This age range aligns with the definition of young people by the World Health Organization [34]
		Studies with mixed age or disease groups were included if we were able to check if quotes were from people who met our criteria
		HCPs^b^, ie, doctors, nurses, and allied HCPs, providing care for young people with T1DM
	**Phenomenon of interest**	
		Studies that addressed experiences and views of young people with T1DM and HCPs of using social media platforms for self-management. As social media platforms, we considered messaging platforms (eg, WhatsApp), mainstream social networks (eg, Facebook, Instagram, Twitter, and Reddit), and disease-specific web portals and online communities (eg, diabetes.uk [35])
		Studies or data from young people and their HCPs were included if they shared their views and perceptions about social media platforms for T1DM self-management regardless of whether they had personal experience of using it as such
	**Context**	
		Any health care context
		This will include self-management activities that young people undertook outside of clinic settings in the context of their daily lives
	**Study type**	
		Any study with qualitative data
		For example, studies reporting findings from interviews or ethnographic research, survey studies reporting free-text comments from questionnaires, studies qualitatively analyzing posts from social media platforms, and mixed methods studies presenting qualitative data or findings (eg, a randomized controlled trial with a nested qualitative process evaluation)
	**Publication type**	
		Original research published in peer-reviewed journals
	**Language**	
		English
	**Time of publication**	
		2012-current
**Exclusion criteria**		
	**Population**	
		People outside the 10-24–year age range or without a diagnosis of T1DM
		Informal caregivers or parents; HCPs providing T1DM care solely for adults
	**Phenomenon of interest**	
		Experiences and views about self-management of T1DM but without references to the role of social media within that, or about social media in general but not in relation to T1DM self-management
	**Context**	
		None
	**Study type**	
		Study only reporting quantitative data (eg, survey studies without analysis of free text comments)
	**Publication type**	
		Reviews, books, conference papers, opinion pieces, commentaries, and gray literature
	**Language**	
		Other languages
	**Time of publication**	
		<2012

^a^Type 1 diabetes mellitus.

^b^Health care professional.

#### Screening

At least 2 reviewers independently conducted 2 rounds of screening (round 1: title or abstract [YM, LH, and KL]; round 2: full text [YM and KL]) to assess papers against the criteria in Table 1. Discrepancies between reviewers were solved through discussion.

### Quality Assessment

We used the Critical Appraisal Skills Programme Qualitative Studies Checklist [36] to assess the quality of included qualitative studies (consisting of 10 criteria) and the Mixed Methods Appraisal Tool (MMAT; 17 relevant criteria) [37] for mixed methods studies. Each study was assessed independently by 2 researchers (YM and KL), with disagreement resolved through discussion. We reported how many criteria each study met as an indicator of their quality. In line with methodological guidance [36,37], we did not remove or assign lower importance to quotations from lower-quality studies, but left it to readers to judge the accuracy of the quotations in reflecting the participants’ viewpoints and perceptions.

### Data Extraction and Synthesis

For objective 1, we extracted information on publication (authors, year of publication, and country), study method (study design, recruitment setting, and data collection), study population (sample size, age, gender, ethnicity, and social media experience), and social media platform (name and type, target users, and purpose). We synthesized information using counts and percentages.

For objective 2, we extracted and thematically synthesized all text labeled as “findings” or “results,” as well as related participants’ quotes [29]. We also extracted participants’ sex and age, if reported at quote level. One researcher (YM) extracted data for all studies, with another (KL) doing this in duplicate for a random 20% (n=3) of studies. Data were exported to NVivo (Lumivero) for further thematic synthesis in 3 steps [29]. First, line-by-line coding in duplicate by 2 researchers (YM and KL) using an inductive approach—the researchers extracted and tabulated all quotes and applied codes that reflected the meaning of the quote. The researchers met regularly to discuss and compare codes, solving disagreements through discussion and with input from another researcher (SvdV) where needed.

Second, developing descriptive themes—once no new codes were identified and data saturation was achieved, 2 researchers (YM, and KL) grouped the final codes into descriptive themes based on their differences or similarities and underlying relationships.

Third, generating analytical themes through a series of discussions within the team to explore how the descriptive themes related to our research aim and objectives, while endeavoring a novel interpretation and insights that expanded beyond simply summarizing the findings of individual-included studies.

## Results

### Overview

**Figure 1:**
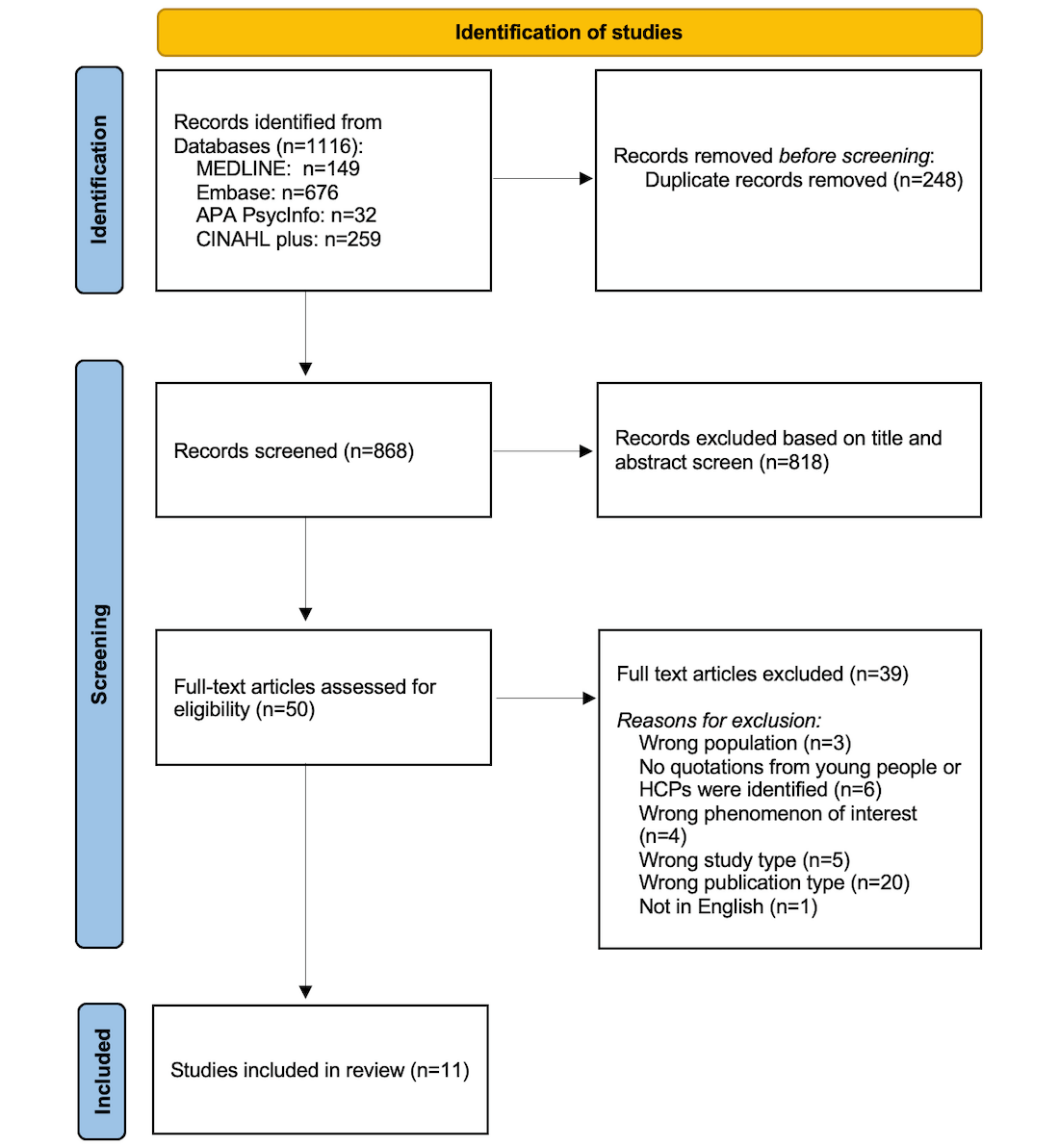
PRISMA flow diagram of the study selection process. HCP: health care professional; PRISMA: Preferred Reporting Items for Systematic Reviews and Meta-Analyses.

Figure 1 shows our search yielded 1116 studies, of which we ultimately included 11 [22,23,38-46]. The main reason for excluding papers based on full text was “wrong publication type” (n=20).

### Study Characteristics

The characteristics, aim, and main results of the included studies were summarized in Multimedia Appendix 2. Of the 11 included studies, the majority were qualitative studies [22,23,38-40,43-46] and conducted in the United States [22,39,41,45,46]. All used content analysis. Most studies focused on young people with T1DM, with only 1 focusing on HCPs [43]. A total of 4 studies used moderated platforms [41-43,46]. All studies met most quality criteria, except 1 that failed to meet 3 out of 10 criteria [38]; Multimedia Appendix 3 contains our detailed quality assessment for each study.

### Synthesis

**Figure 2:**
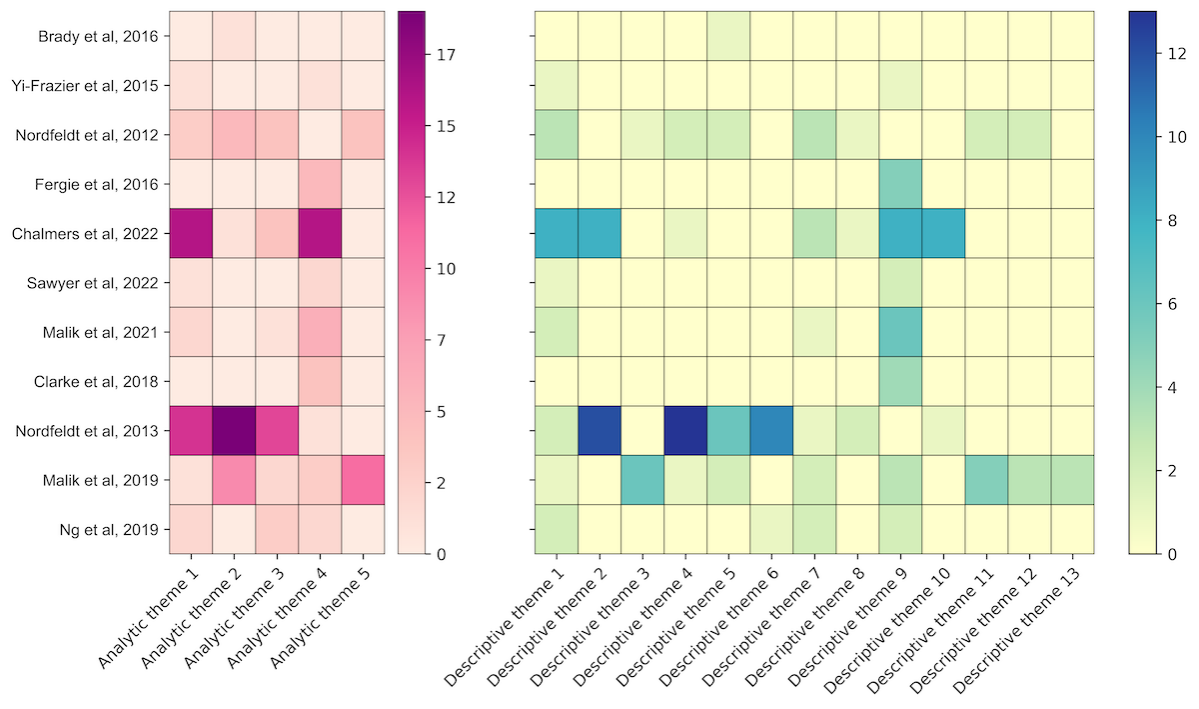
Heat map visualizing the extent to which studies contributed to analytic and descriptive themes. Darker colors indicate more substantial contributions and the numbers beside the color bars refer to the number of quotations coded under each theme [22,23,38-46].

Table 2 shows how we identified 13 descriptive themes that contributed to 5 analytic themes, with Figure 2 showing to what extent studies contributed to each of the themes (see Multimedia Appendix 4 for the underlying study-level information).

**Table 2:** Analytic and descriptive themes with additional illustrative quotations.

Analytic and descriptive themes		Additional illustrative quotations
**Analytic theme 1: differences in how young people interact with social media (differences in how young people with T1DM^a^ interacted with social media, including different levels of engagement and willingness to disclose information about their condition).**		
	Descriptive theme 1: passive versus active user engagement	“I also read blogs, but don’t post anything myself.” [HCPs^b^], [43] “I’ve made posts about going to camp with other diabetics or that I’m with my ‘dia-besties.’” [Female participant aged 16 years], [39]
	Descriptive theme 2: levels of T1DM disclosure	“A girl I went to middle school with has [information about the fact that she has diabetes in] her Instagram handle. some people have their handles as their real names and then have type 1 in their bio.” [Female participant aged 18 years], [39] “[Y]ou see most websites, if you’re creating a website which you don’t want everybody to see...only just friends, then you can arrange it with passwords” [44] “People don’t understand. Most people think you have to be overweight to have diabetes and they’re like, ‘How do you have diabetes? You’re not fat’” [Female participant aged 14 years], [39]
**Analytic theme 2: characteristics of social media platforms that influence their use and uptake for T1DM self-management (characteristics of social media that influenced their use and uptake for T1DM self-management, including easier access to information and peer support, platform design, and trustworthiness).**		
	Descriptive theme 3: easier access to information and peer support	“The advantages are that it is handy if you need to look something up, accessible information, and that it is easy to search” [HCPs], [43] “I get a lot of emails and it’s really hard to distinguish what is spam and what is not [since] sometimes it doesn’t go into the spam box” [22]
	Descriptive theme 4: platform design	“I feel like a social media page would be a lot better because depending on how it would be set up, it would be organized” [22] “I want like...I want it to be colourful and funny...” [Girls, 10-11 years], [44]
	Descriptive theme 5: trustworthiness	“The website here looks serious, if it should be some strange person, or for instance a paedophile, I don’t think that he would enter a diabetes website and look there. I rather think that those who enters a diabetes website are those interested in diabetes as a subject” [Girls, 14-15 years], [44] “If it comes across as reasonable and educational, you know, you kind of trust in it. rather than someone who types ‘lyk dis’” [18-25 years], [38] “If you feel certain about what kind of website it is, who’s behind it, then you can trust that there’s no false things there” [Girls, 12-13 years], [44]
**Analytic theme 3: social media as a source of information (social media had the potential to cater to diverse information needs and provided an avenue to learn from peers and educate others).**		
	Descriptive theme 6: catering for diverse information needs	“And there is news, what happens in the body, sexuality, and lots of different things...about insulin and medical devices, and about food and how you’re affected by things...what you ought to think about, not to smoke, and with diabetes, don’t drink and such stuff” [Girls, 14-15 years], [44]
	Descriptive theme 7: learning from peers’ experience	“It was so helpful to be able to ask questions and see the questions asked by others to learn from their experiences and gain knowledge of how others manage their diabetes” [Female participant aged 23 years old], [42]
	Descriptive theme 8: educating others	“School and preschool staff can visit the site to prepare themselves prior to our visits” [HCPs], [43]
**Analytic theme 4: impact on young people’s coping and emotional well-being (positive and negative effects social media could have on young people’s coping and emotional well-being).**		
	Descriptive theme 9: emotional support from peers	“Have friends who you know are also going through the same problems and who you know understand what you are feeling” [Female participant aged age 22], [23] “I think if you have something as big as diabetes in common then like you could probably bond really fast” [22]
	Descriptive theme 10: humor and hope	“I just post like jokes and relatable stuff for diabetics...When it comes to my diabetic page, I just hope it makes someone laugh...It’s just a bunch of memes.” [Female participant aged 14 years], [39] “It would be fun to know just, yes, that there will be the remedy for diabetes within 15 years and...it is...they have been talking a lot about finding a certain remedy to regain your insulin production” [Boys, 14-15 years], [44]
**Analytic theme 5: Impact on support from and relationship with HCPs^b^ and the health care service (how social media might affect the support from and relationship with HCPs and the health care service) support from and relationship with HCPs and the health care service).**		
	Descriptive theme 11: more direct support from HCPs	“If it’s a question that affects your care during those three months [between clinic visits], or it does something to help what you’re doing within diabetes, then that could be helpful because you get the answer then and not have to wait” [22]
	Descriptive theme 12: better HCP-patient relationships	“By doing so I learn how the patients think, which can be useful in working with them (HCPs) [43], [Through social media] they would know more about me, and what I like and what kind of foods I like, so that could also factor into insulin pump settings” [22]
	Descriptive theme 13: potential privacy concerns	“In a DM [conversation] it’s only going to me...[but] I feel like if it was me and [care team member] in a DM, I could add anyone at any time, or [the care team member] could add anyone at any time, and then it’s no longer private” [22] “I think it would be a little weird [engaging over social media] at first” [22]

^a^T1DM: type 1 diabetes mellitus.

^b^HCP: health care professional.

### Analytic Theme 1: Differences in How Young People Interact With Social Media

#### Descriptive Theme 1: Passive Versus Active User Engagement

Young people exhibited different levels of engagement on social media. Some could be categorized as “content consumers” or “lurkers” who tended to be more passive observers [39,43]. They might consume content, such as reading blogs and following forums, to stay informed about the others’ experiences, but they rarely contributed content or engaged with the other users [39,43]. This might be due to a lack of time, having other priorities, or not being interested in posting [41,42,44]. For example [39],

There’s nothing for me to like post about, I don’t do like blood sugar updates on my story or anything.

Male participant aged 13 years

On the other hand, there were “active participants” who actively contributed to the social media community. They regularly shared content related to their diabetes journey, interacted with T1DM peers, offered insights to raise awareness, and fostered connections within the diabetes community [39]. Some “active participants” also worked as “role models” to motivate fellow users by showing a positive attitude toward T1DM management and sharing how they combatted their condition [44,45]. For example [39],

[I]t’s second nature. That’s the mood I would want [my followers] to get out of [my posts]. Show it who’s boss.

Male participant aged 16 years

#### Descriptive Theme 2: Levels of T1DM Disclosure

Young people expressed different opinions toward diabetes disclosure on social media. Some were comfortable being open and outspoken about having T1DM, freely disclosing their condition in their posts and even profile descriptions on public-facing platforms such as Instagram (Meta Platforms) and Twitter (Twitter, Inc), thus appearing to consider it part of their online identity [39]. For example [39],

When you have an Instagram [account], you set your profile...and I put that I’m diabetic on there.

Female participant aged 18 years

Others selectively shared their T1DM-related activities in more inclusive platforms such as Facebook (Meta Platforms) group and Snapchat (Snap Inc), appreciating the features that allowed them to choose audiences or block unwanted viewers to keep themselves safe and protected from unwanted strangers accessing personal information [39,44]. For example [39],

Snapchat has a feature where you can block people from seeing your story and stuff, so I am comfortable [discussing T1D] because I can control who sees what.

Additionally, some young people chose not to share their T1DM-related activities possibly due to previous bad experiences or concerns about potential misunderstanding or judgment from others [39]. For example, one female participant aged 18 years explained [39],

I have always gotten bullied for it (T1D), so I keep it on the down low on social media.

### Analytic Theme 2: Characteristics of Social Media Platforms That Influence Their Use and Uptake for T1DM Self-Management

#### Descriptive Theme 3: Easier Access to Information and Peer Support

Social media were perceived by young people of T1DM and HCPs to be easier and more timely means of communication and to have quicker access to information compared with traditional methods such as email and phone calls [22,43]. For example, unlike the complexities of searching through emails and distinguishing spam, it was “just at the tip of your fingers” [22] to flip through information and communicate with others.

Social media also provided an alternative online channel for seeking peer support compared with in-person interactions. This online support complemented face-to-face interactions, offering individuals a sense of autonomy to access social support whenever they felt ready and comfortable [41]. At the same time, however, others felt in-person meetings could offer a chance to better understand personalities and foster deeper connections [41].

#### Descriptive Theme 4: Platform Design

The platform’s user interface design and how content was presented influenced young people’s and HCPs’ choice of using social media in T1DM self-management [22,43,44]. Young people and HCPs expressed a preference for well-organized platforms that simplified the process of finding specific information [22]. Platforms that appeared messy and overwhelming with excessive information might deter users’ exploration and engagement [43,44]. Additionally, young people preferred regularly updated content that aligned with their needs—they found it difficult to feel engaged or interested if platforms did not offer anything new or relevant to their experiences [39,44]. Younger participants further expressed a preference for colorful platforms that provided age-specific information on a wide range of topics [39,44].

#### Descriptive Theme 5: Trustworthiness

Trustworthiness was another important part that affected young people’s use and HCPs’ recommendation of social media for T1DM self-management.

First, young people with T1DM and HCPs tended to trust platforms with well-presented facts [44], using formal and professional language rather than casual language such as “lyk dis” (18-25 years) [38].

Second, young people were more likely to use platforms that were recommended by sources they perceived as reliable, such as HCPs and peers [22,43], while HCPs felt more comfortable offering information via sites that were developed with or moderated by HCPs to ensure accurate information was given [43]. One HCP explained [43],

What feels safe for me...is that the information they can read here is the information we have provided. We know that we have critically reviewed it together.

Third, young people trusted transparent platforms where they knew who was allowed to join and use the platforms as users. Otherwise, they might feel unsafe or concerned about interacting with malicious or deceptive individuals [44].

### Analytic Theme 3: Social Media as a Source of Information

#### Descriptive Theme 6: Catering for Diverse Information Needs

Social media could provide diverse diabetes-related information, such as food and sports tips, insulin and pump instructions, information about drugs and medicine, advice on healthy habits (eg, abstaining from smoking or drinking alcohol), and insights on long-term complications and future expectations about life quality [39,44]. Moreover, social media provided information tailored to different groups of people, such as age-specific information for children and young people and diabetes management guidance tailored to the needs of caregivers and school staff [44].

#### Descriptive Theme 7: Learning From Peers’ Experience

Social media also served as a valuable platform for young people with T1DM to learn from the experiences of their peers who openly discussed different treatment options and gave advice based on their experience [39,43]. This real-life experience complemented the more standardized advice from HCPs and clinical services [39,44], bringing pros and cons of treatment to life. As such, it better informed young people’s understanding of different treatment options [22,40,41,44]. One female participant aged 18 years explained [42],

...through the website and seeing how other people manage their health to see what could work better for me.

#### Descriptive Theme 8: Educating Others

Social media offered opportunities for others to learn and stay up-to-date about T1DM. For example, caregivers and school staff could equip themselves with knowledge involving T1DM management to provide more appropriate and informed support to young people with T1DM [43]. Additionally, the knowledge shared on certain types of public-facing social media (eg, YouTube videos and hashtag campaigns on Instagram and Twitter) played a vital role in raising public awareness and reducing misunderstandings [44], such as the misconception that T1DM was associated with being overweight as shown in the quote *“*...Most people think you have to be overweight to have diabetes.” (female participant aged 14 years) [39].

### Analytic Theme 4: Impact on Young People’s Coping and Emotional Well-Being

#### Descriptive Theme 9: Emotional Support From Peers

T1DM self-management has the potential to be an overwhelming process, often leading to mental health issues, such as anxiety and depression [10,48]. While social media could be a source of emotional support to help them manage these [22,40,42,46], there were examples in the literature of it being associated with negative feelings, for example, diabetes-related stigma [39] and sometimes increasing feelings of loneliness [42].

First, social media provided a platform for young people with T1DM to openly share their feelings and seek comfort and understanding from other users [23]. When they did so, it was very common to receive positive and encouraging responses [39]. These positive responses from their peers could validate their condition and uplift their spirits, making them realize they were not alone in the journey of managing T1DM and gradually accepting the condition as “second nature” [39]. For example [41],

Just feeling like you’re not the only one going through it...and that you can still lead like a normal life and still have diabetes.

At the same time, however, others felt that despite the positive feedback they received, those without T1DM could not fully comprehend their challenges [39]. This sense of disconnection further intensified their feelings of isolation [39]. One female participant aged 17 years explained [39],

[Other social media users] would be like, “Do your best” or “You’re doing wonderful!” But a lot of people don’t know, and I feel like that makes you feel a little bit more alone.

Female participant aged 16 years

The conflicting views above highlighted the importance of emotional support from peers with shared experiences and who truly understood the challenges. This shared understanding could give young people a sense of belonging and normalization, creating a safe space where they could freely express their emotions and struggles. Furthermore, shared experiences could easily foster strong bonds and friendships with others [22]. For example, young people tended to use social media to reconnect with peers after attending a face-to-face diabetes camp [45]. This was particularly important for young people who lacked family support, as it could provide supplementary information and emotional help [40,44].

#### Descriptive Theme 10: Humor and Hope

Humor and hope were 2 important strategies that young people used to cope with T1DM [39,44]. By joking about their condition on social media, they could reduce the stress associated with it and treat it as “less of a big deal and kinda normalize it” (male participant aged 15 years) [39]. Additionally, some young people used humor to defend themselves against potential bullying and negative comments. For example, 1 person explained, “I just have to eliminate that chance [of bullying] by like making fun of me before they do” [39]. Moreover, some young people posted jokes and humor and had a positive outlook on social media to inspire their peers to take on a more optimistic approach to deal with their long-term conditions [39]. Finally, young people also expressed hopes of a cure for T1DM on social media [44]. This outlook could fill them with hope and excitement, igniting a sense of optimism for the future.

### Analytic Theme 5: Impact on Support From and Relationship With HCPs and the Health Care Service

#### Descriptive Theme 11: More Direct Support From HCPs

Social media changed the way how young people with T1DM received support from HCPs [22,43]. In some cases [22], young people reported that their parents communicated with HCPs about their diabetes and felt their parents were not fully informed about their condition. Social media enabled young people with T1DM to communicate directly with their HCPs, promoting greater engagement in self-management and empowering them to take control of their condition [22]. For example [22],

My parents are normally the ones who would talk with the doctors and stuff. and so with social media it would be like I’m taking more control over what’s happening.

Young people also valued the timeliness of communication via social media in between clinic visits. For example, when wanting to contact HCPs, young people with T1DM could send direct messages to their HCPs, rather than going to clinics, sending emails that were lost or never replied to, or trying to get in contact through a phone [22,43].

#### Descriptive Theme 12: Better HCP-Patient Relationships

Engaging with HCPs through social media offered the potential to cultivate a more personalized HCP-patient relationship [22,43]. By sharing insights with HCPs about their lifestyle and preferences, HCPs could better understand young people’s conditions and adopt a more patient-centered approach to care. Young people with T1DM and HCPs believed this resulted in better and more tailored support [22,43]. For example, “you would probably get to know each other a bit more and you would be more knowledgeable about things going on” [22].

#### Descriptive Theme 13: Privacy Concerns

Despite the benefits mentioned above, some young people felt awkward when they engaged with their HCPs through social media [22]. They expressed privacy concerns about the potential inspection and judgment if their HCPs were a part of their personal social media platforms or if their HCPs inadvertently invited someone they did not know into their private conversation, potentially leaking their personal information to others [22]. For example [22],

...it might be awkward at the same time, like, if your doctor’s following you on Instagram and they can see everything you’re posting and all that stuff.

## Discussion

### Summary of Findings

This synthesis systematically reviewed the qualitative data on experiences and views of young people with T1DM and their HCPs regarding the use of social media for self-management. We included 11 studies in our synthesis, 10 of which focused on young people with T1DM. All used content analysis and met most quality criteria.

A total of 5 analytical themes and 13 descriptive themes were yielded by the synthesis. It revealed varying levels of engagement and comfort among young people with T1DM in using social media for self-management, with greater levels of trustworthiness given to professionally designed platforms recommended by HCPs or peers. Social media facilitated young people’s access to a wide range of information and peer support and enabled easier and more direct communication with HCPs. Privacy and safety concerns were the main barriers preventing the use of social media for T1DM self-management.

### Relation to Other Studies

Several of our findings were in accordance with previous research on the role of social media for self-managing T1DM in young people. For example, consistent with other reviews [8,14,27,49,50], we identified that young people with T1DM used social media to enhance their diabetes management knowledge and skills and to provide and receive emotional peer support. Similarly, we confirmed that social media could complement traditional health care services by providing direct communication with HCPs, complemented with tailored insights from online peers. Beyond these studies, we additionally identified the supplementary role of social media in complementing family and face-to-face support [41]. Overall, our findings reinforced the established understanding of the supplementary supportive potential of social media for T1DM self-management.

In contrast to previous research [8,27,49], we did not find many concerns related to misinformation. This may be explained by young people’s trust in the experiences and tips shared by their online peers [22,43]. However, these individualized experiences may not be a suitable source for everyone and may contain information that is not necessarily accurate [22,37]. Furthermore, privacy and safety concerns were the main risks identified in the synthesis, often preventing young people from using social media [22,39]. These risks were linked to disclosing excessive personal information online, which in turn might lead to things such as online bullying, harassment, and encountering malicious individuals [22,27,44]; these risks and issues are common in any online activity and can be minimized by using social media safely [27].

Finally, our synthesis revealed novel insights into HCPs’ role in using social media for T1DM self-management. We found that HCPs encouraged patients to connect with peers on social media to gain diverse perspectives on T1DM treatment and to use HCP-moderated online platforms, while other research found that HCPs might advise against patients’ social media use [50,51]. Also, where previous reviews and studies indicated HCP involvement in self-management could improve HCP-patient relationships and health care delivery more generally [8,50-52], our synthesis found more diverse patient attitudes, ranging from beliefs that HCP involvement would enable tailored support to worries about potential scrutiny of their social media posts.

### Implications for Practice and Research

#### Implications for Social Media Platforms and HCPs

The supplementary role of online peers’ experiences identified in our synthesis, which was confirmed by other studies [8,14,27,49,50], implies that HCPs should consider integrating customized peer-to-peer mechanisms into traditional services to provide better health care. For example, moderated social media online forums and Facebook groups such as Diabetes UK [35] and Diabetes Yes [42] have created online communities that provide professional-reviewed knowledge and real-life experiences from peers that complement medical care. HCPs could recommend that their patients access these platforms to get information and peer support between clinic visits. Meanwhile, they could participate in moderated online groups to communicate with and support their patients.

However, the reliability and applicability of information and experiences exchanged online cannot be assured [22,37], social media platforms and HCPs should educate young people on how to identify credible online health information about their T1DM, for example by providing evidence-based knowledge and contrasting it against misleading content [22,43].

Furthermore, our synthesis showed that young people have different comfort and engagement levels in sharing their T1DM-related content online. This may warrant further refinement of available functionality within social media platforms to give users more control over what they share with whom, such as features allowing selective audiences or restricted profile access.

#### Implications for Health Policy Makers

Our synthesis identified that young people had privacy and safety concerns about using social media and involving HCPs for T1DM self-management [22,39,44]. Existing guidelines [27,53,54] provided a broad and comprehensive framework for safe social media use and appropriate HCP involvement, but these did not incorporate patient and HCP input, and may be outdated given the rapidly evolving social media landscape. Furthermore, protections for patients regarding inappropriate HCPs’ involvement were insufficiently addressed.

Recommendations could be enhanced by developing updated and evidence-based guidelines that incorporate input from patients and HCPs. Informed consent procedures could be added to existing guidelines to protect patients by (1) informing patients on the risks and best practices of using social media for T1DM self-management; (2) emphasizing patients can opt out of HCPs’ involvement in their social media T1DM self-management; (3) providing clear conduct guidelines for HCPs; and (4) establishing formal reporting channels for patients’ concerns over uncomfortable interactions, boundary violations, or privacy issues.

#### Implications for Future Research

The participants in the included studies were mostly social media users; only 1 study in our synthesis [22] included a small proportion of participants who did not have social media experience. This may have introduced a potential bias toward more positive views, especially as several studies interviewed young people involved in developing a social media platform. To gain a more holistic perspective and inform platforms and guidance suitable for a broad range of young people with T1DM, future research could include input from nonsocial media users.

The first-order qualitative data included in our synthesis consisted of participants’ quotations about their experiences and opinions. Future studies could analyze young people’s social media posts to gain additional insights into their actual (instead of reported) online behaviors, attitudes, and topics of interest.

### Limitations

One limitation of our synthesis came from the exclusion of non-English studies, which means we may have missed insights from non-English studies on this topic. Widening the scope beyond English could enrich our findings by uncovering different cultural perspectives from non-English speaking contexts that were not captured in our synthesis.

Another limitation arose from solely analyzing qualitative data from original studies published in peer-reviewed journals. This excluded potentially valuable inputs from gray literature and text from social media platforms that may have provided additional real-world practices, patient experiences, and emerging trends relevant to this topic. Incorporating these wider sources in future analyses could lead to a more comprehensive synthesis of diverse qualitative evidence.

### Conclusions

Our synthesis identified the experiences and views of young people and HCPs using social media for T1DM self-management. It reinforced social media’s role in providing peer support, supplementary information, and emotional support. In addition, privacy and safety concerns were identified as key barriers preventing young people from engaging with social media for support and information.

The synthesis suggests we should consider leveraging social media’s peer support capabilities to augment traditional services for young people with T1DM. However, patients may have privacy concerns about the HCPs’ involvement in their online activities. This warrants an update of existing guidelines to help young people use social media safely for self-managing their diabetes.

## Appendix

Multimedia Appendix 1 Search strategy.

Multimedia Appendix 2 Characteristics, aim, and main results of included studies.

Multimedia Appendix 3 Quality assessment.

Multimedia Appendix 4 Themes identified in each study.

## Abbreviations

HCP: health care professional
MMAT: Mixed Methods Appraisal Tool
PRISMA: Preferred Reporting Items for Systematic Reviews and Meta-Analyses
T1DM: type 1 diabetes mellitus
