# Supplementary material for: Experiences and Views of Young People and Health Care Professionals of Using Social Media to Self-Manage Type 1 Diabetes Mellitus: Thematic Synthesis of Qualitative Studies
Source: JMIR Pediatr Parent. 2024 May 29;7:e56919. doi: 10.2196/56919 (PMC11170052; doi:10.2196/56919)
Supplement: Multimedia Appendix 1 [file pediatrics_v7i1e56919_app1.docx]

**Table S1.** Ovid MEDLINE(R) ALL (1946 to February 15, 2023).

| 1 | (Insulin-Dependent Diabetes Mellitus or Juvenile-Onset Diabetes Mellitus or IDDM or Juvenile-Onset Diabetes or Juvenile Onset Diabetes or Sudden-Onset Diabetes Mellitus or Type 1 Diabetes Mellitus or Insulin-Dependent Diabetes Mellitus 1 or Insulin Dependent Diabetes Mellitus 1 or Type 1 Diabetes or Autoimmune Diabetes or Brittle Diabetes Mellitus or Ketosis-Prone Diabetes Mellitus).ab,ti. |
| --- | --- |
| 2 | (online forum* or diabetes online communit* or Facebook or Twitter or TikTok or Youtube or Instagram or Pinterest or "google plus" or WhatsApp or Flickr or Livejournal or LinkedIn or Tumblr or skype or WeChat or QQ or QZone or Weibo or Douban or Reddit).ab,ti. |
| 3 | Diabetes Mellitus, Type 1.mp. or exp Diabetes Mellitus, Type 1/ |
| 4 | social media.mp. or exp Social Media/ |
| 5 | exp "Internet Use"/ |
| 6 | online social networking.mp. or exp Online Social Networking/ |
| 7 | blogging.mp. or exp Blogging/ |
| 8 | 4 or 5 or 6 or 7 |
| 9 | 2 or 8 |
| 10 | 1 or 3 |
| 11 | 9 and 10 |
| 12 | limit 11 to (english language and yr="2012 -Current") |

**Table S2.** Embase (1980 to 2023 week 06)**.**

| 1 | insulin dependent diabetes mellitus.mp. or exp insulin dependent diabetes mellitus/ |
| --- | --- |
| 2 | (Insulin-Dependent Diabetes Mellitus or Juvenile-Onset Diabetes Mellitus or IDDM or Juvenile-Onset Diabetes or Juvenile Onset Diabetes or Sudden-Onset Diabetes Mellitus or Type 1 Diabetes Mellitus or Insulin-Dependent Diabetes Mellitus 1 or Insulin Dependent Diabetes Mellitus 1 or Type 1 Diabetes or Autoimmune Diabetes or Brittle Diabetes Mellitus or Ketosis-Prone Diabetes Mellitus).ab,ti. |
| 3 | 1 or 2 |
| 4 | social media.mp. or exp social media/ |
| 5 | online social network.mp. or exp online social network/ |
| 6 | "internet use".mp. or exp "internet use"/ |
| 7 | blogging.mp. or blogging/ |
| 8 | 4 or 5 or 6 or 7 |
| 9 | (online forum* or diabetes online communit* or Facebook or Twitter or TikTok or Youtube or Instagram or Pinterest or "google plus" or WhatsApp or Flickr or Livejournal or LinkedIn or Tumblr or skype or WeChat or QQ or QZone or Weibo or Douban or Reddit).ab,ti. |
| 10 | 8 or 9 |
| 11 | 3 and 10 |
| 12 | limit 11 to (english language and yr="2012 -Current") |

**Table S3.** APA PsycInfo (1806 to February week 1 2023).

| 1 | (Insulin-Dependent Diabetes Mellitus or Juvenile-Onset Diabetes Mellitus or IDDM or Juvenile-Onset Diabetes or Juvenile Onset Diabetes or Sudden-Onset Diabetes Mellitus or Type 1 Diabetes Mellitus or Insulin-Dependent Diabetes Mellitus 1 or Insulin Dependent Diabetes Mellitus 1 or Type 1 Diabetes or Autoimmune Diabetes or Brittle Diabetes Mellitus or Ketosis-Prone Diabetes Mellitus).ab,ti. |
| --- | --- |
| 2 | (online forum* or diabetes online communit* or Facebook or Twitter or TikTok or Youtube or Instagram or Pinterest or "google plus" or WhatsApp or Flickr or Livejournal or LinkedIn or Tumblr or skype or WeChat or QQ or QZone or Weibo or Douban or Reddit).ab,ti. |
| 3 | social media.mp. or exp Social Media/ |
| 4 | online social networks.mp. or exp Online Social Networks/ |
| 5 | internet usage.mp. or exp Internet Usage/ |
| 6 | exp Online Behavior/ or online behavior.mp. |
| 7 | online community.mp. or exp Online Community/ |
| 8 | blog.mp. or exp Blog/ |
| 9 | 3 or 4 or 5 or 6 or 7 or 8 |
| 10 | 2 or 9 |
| 11 | 1 and 10 |
| 12 | limit 11 to (english language and yr="2012 - 2023") |

**Table S4.** CINAHL plus (February 16, 2023).

| 1 | (MH "Diabetes Mellitus, Type 1+") OR "type 1 diabetes" |
| --- | --- |
| 2 | AB Insulin-Dependent Diabetes Mellitus or Juvenile-Onset Diabetes Mellitus or IDDM or Juvenile-Onset Diabetes or Juvenile Onset Diabetes or Sudden-Onset Diabetes Mellitus or Type 1 Diabetes Mellitus or Insulin-Dependent Diabetes Mellitus 1 or Insulin Dependent Diabetes Mellitus 1 or Type 1 Diabetes or Autoimmune Diabetes or Brittle Diabetes Mellitus or Ketosis-Prone Diabetes Mellitus |
| 3 | TI Insulin-Dependent Diabetes Mellitus or Juvenile-Onset Diabetes Mellitus or IDDM or Juvenile-Onset Diabetes or Juvenile Onset Diabetes or Sudden-Onset Diabetes Mellitus or Type 1 Diabetes Mellitus or Insulin-Dependent Diabetes Mellitus 1 or Insulin Dependent Diabetes Mellitus 1 or Type 1 Diabetes or Autoimmune Diabetes or Brittle Diabetes Mellitus or Ketosis-Prone Diabetes Mellitus |
| 4 | (MH "Social Media+") OR "social media” |
| 5 | (MH "Online Social Networking") OR "online social network*" OR "online social network" |
| 6 | (MH "Blogs") OR "blog” |
| 7 | AB online forum* or diabetes online communit* or Facebook or Twitter or TikTok or Youtube or Instagram or Pinterest or "google plus" or WhatsApp or Flickr or Livejournal or LinkedIn or Tumblr or skype or WeChat or QQ or QZone or Weibo or Douban or Reddit |
| 8 | TI online forum* or diabetes online communit* or Facebook or Twitter or TikTok or Youtube or Instagram or Pinterest or "google plus" or WhatsApp or Flickr or Livejournal or LinkedIn or Tumblr or skype or WeChat or QQ or QZone or Weibo or Douban or Reddit |
| 9 | 1 OR (2 OR 3) |
| 10 | (4 OR 5 OR 6) OR (7 OR 8) |
| 11 | 9 AND 10 |
