# Supplementary material for: Experiences and Views of Young People and Health Care Professionals of Using Social Media to Self-Manage Type 1 Diabetes Mellitus: Thematic Synthesis of Qualitative Studies
Source: JMIR Pediatr Parent. 2024 May 29;7:e56919. doi: 10.2196/56919 (PMC11170052; doi:10.2196/56919)
Supplement: Multimedia Appendix 2 [file pediatrics_v7i1e56919_app2.docx]

| **Author**  **Publication year**  **Country** | **Study methods** | | | **Study population** | | | | | **Social media platforms** | | **Study quality ^b^** | **Aim** | **Main results** |
| --- | --- | --- | --- | --- | --- | --- | --- | --- | --- | --- | --- | --- | --- |
|  | *Study design* | *Recruitment strategy and setting* | *Data Collection* | *Sample size ^a^* | *Age (years)* | *% Female gender* | *Ethnicity* | *% with social media experience* | *Name*  *Type* | *Specifically targeted at young people with T1DM?*  *Specific purpose?* |  |  |  |
| Ng et al, [42]  2019  Australia | Mixed methods with a qualitative content analysis | Online and ongoing recruitment through social media (e.g., Facebook and Twitter) | Survey using Likert scales and open-ended questions | 34 | 18 – 35;  Mean ± SD (27 ± 5) | 82% | NR | 100 | Diabetes Yes  Mobile-optimized website and closed Facebook group, moderated by one researcher with lived experience of T1DM. Weekly discussion topics posted by moderator | Yes  Yes, provide peer support | 16/17 | Examine feasibility and acceptability of the Diabetes YES program | Emotional support from peers regarded as having highest benefit. Peer support engagement through fb was maintained, visits to website declined sharply. Most quotes seem to support the need to see how others manage symptoms so they can learn from each other and validate their own feelings. Peer support improved well-being |
| Malik et al, [22]  2019  USA | Qualitative content analysis | Mail and phone to recruit from hospital diabetes clinics | Focus groups with semi-structured interviews | 45 | Mean ± SD (15.9 ± 1.7) | 42% | 78% non-Hispanic white | 93 | Social media in general | No  No | 10/10 | To explore the feasibility of using social media as a tool to collaboratively manage T1D with the T1D care team | Seven major topics identified:  (1) improved communication outside of clinic visits to optimize diabetes management, (2) independence in diabetes self-management, (3) connection to other youth with diabetes for additional diabetes support, and (4) delivery of more personalized care. 5) ensure patient privacy, (6) maintain professional nature of provider-patient relationship, and (7) recognize that social media is not currently used for medical care by youth with diabetes |
| Nordfeldt et al, [44]  2013  Sweden | Qualitative content analysis | Posted information letter to recruit from county hospital | Focus groups with interview guides | 24 | 10-17 | 46% | NR | 100 | Social media in general | No  No | 10/10 | The purpose of this study was to understand information-seeking behaviors, Internet use and social networking online in adolescents with type 1 diabetes (T1DM) | Three main categories were identified: (1) Aspects of security: which consists of three sub-categories: seriousness, integrity and identity. (2) updating: which consists of three sub-categories: news value, facts, and eye-catching. (3) plainness: which consists of three sub-categories: layout, content and congeniality. These categories and sub-categories gave significant information about how to enhance information retrieval and peer contacts related to T1DM |
| Clarke et al, [23]  2018  Australia | Qualitative content analysis | Facebook and Twitter adverts, announcements on websites of local diabetes advocacy agencies, referral by staff of transition services | Focus groups with semi-structured interviews | 31 | 18-30; Mean ± SD (22 ± 3) | 84% | NR | NR | Online peer support platforms in general | No  Yes, provide mental health support | 10/10 | To explore the attitudes and feelings of young adults with type 1 diabetes towards mental health and mental health research and the enthusiasm of this cohort for digital mental health interventions | Four Main themes: (1) Attitudes towards mental health and mental health research, (2) Potential barriers to and motivators for participating in mental health research, (3) Strategies for engaging young adults in mental health research, (4) Sources of mental health support |
| Malik et al, [41]  2021  USA | Mixed method with a qualitative content analysis | Hospital settings | Focus groups followed by a questionnaire | 16 | 13-18 | 56.25% | NR | 100 | Instagram  Dedicated Instagram group, moderated by researchers | Yes  Yes, provide peer support | 15/17 | To assess the feasibility and acceptability of a hybrid support group model for adolescents with T1DM. | Support group participation used Instagram as application of photovoice, consistently posting photos. The ability to learn from others and not feel alone was a key reason they enjoyed posting and participating in the support groups. This empowering approach was found to enhance self-reflection and foster meaningful photo discussions on Instagram |
| Sawyer et al, [45]  2022  USA | Qualitative content analysis | Electronic mailing list and social media platforms (e.g., Facebook and Instagram) | Telephone interviews | 21 | Mean ± SD (15 ± 8) | 90.48% | NR | NR | Social media in general | No  No | 9/10 | To identify barriers and strategies to diabetes management as informed by the lived experiences of emerging adults with T1DM | Main themes: barriers: physiology, mental health, environment, lack of support, weather, insurance. Strategy themes: medical technology; access to social support; physical activity |
| Chalmers et al, [39]  2022  USA | Qualitative content analysis | Mail recruitment of people identified from hospital’s medical record. | Semi-structured interviews with interview guide | 35 | Mean ± SD (14.9 ± 1.7) | 48.6% | 60% non-Hispanic White;  31.4% Non-white, non-Hispanic;  8.6% prefer not to say | NR | Social media in general | No  No | 10/10 | To explore adolescents’ experiences and perspectives discussing their T1D on social media | Four affordances of social media associated with adolescents’ experiences related to discussing T1D on social media were identified: identity affordance, cognitive affordance, emotional affordance, and social affordance |
| Fergie et al, [40]  2016  UK | Qualitative content analysis: thematic analysis | Online (e.g., Facebook groups, online forums) and offline recruitment (further/higher education institutions, recommendations from gatekeepers and patients) | Semi-structured interviews | 40 | 18-30 | 50% | NR | 100 | Facebook, YouTube and Twitter | No  No | 9/10 | To gain new insights into how young adults with long-term health conditions engage with health-related user-generated content on social media sites in their day-to-day management of their health. And to identify any barriers that limit users from adopting these technologies to support their health experiences | Young adults showed diverse online activities, ranging from regularly producing and consuming health-related user-generated content to not engaging at all. This research identified three user types: “prosumers,” “tacit consumers,” and “non-engagers.” Offline support experiences were a significant factor in their engagement with diabetes and CMHD-related resources online. Concerns about compromising their identity presentation and adhering to social media conventions were barriers to participating and sharing health-related content online |
| Nordfeldt et al, [43]  2012  Sweden | Qualitative content analysis | Email invitation | Email invitations to write essays describing their experience using the portal | 18  practitioners including doctors, nurses, dietitians, and a social welfare officer | NR | NR | NR | 100 | Diabit  A Web portal moderated by HCPs and patients | Yes  Yes, provide diabetes-related information and communication | 9/10 | To explore practitioners’ perceptions of using an open-access interactive Web portal tailored to young diabetes type 1 patients and their guardians or significant others | Three categories were identified: (1) to use or not to use: refers to if practitioners found the platforms useful in their practices. (2) information center for everyone: embraces the portal as a source of scientifically sound information and advice for patients, guardians, and significant others, as well as for other professionals (3) developing our practice: reflects on what information to give through the portal and how to give it, learning more about patients’ views, and adapting more to patients’ needs |
| Yi-Frazier et al., [46]  2015  USA | Qualitative content analysis | Purposive mail, phone, or in-person recruitment to recruit patients at a large tertiary care hospital | Individual interviews and a focus group | 20 | 14-18;  Mean ± SD (16.4 ± 1.6) | 65% | NR | 100 | Instagram  Dedicated Instagram group, moderated by researchers | Yes  Yes, provide peer support | 8/10 | To assess the feasibility of the intervention platform and categorize the types of diabetes-related photos shared by participants | Five main categories were identified: diabetes care, humor, food, exercise/sports, and life with diabetes. Engaged participants universally reported the project to be a positive experience |
| Brady et al., [38]  2016  UK | Qualitative content analysis. | Online and offline, via internet forums, face-to-face support groups, email lists, and research networks across the UK | Semi-structured interviews | 41 | 18-82; Mean: 50 | 68% | NR | NR | Diabetes-related online forums in general | No  No | 7/10 | to explore how individuals with LTCs assess and trust the peer-sourced health information that they encounter online | Three themes were identified: (1) Collective identity and individual markers of trust. (2) Gaining credibility points: Constructing a knowledgeable identity online. (3) Establishing evidence-based lay knowledge |

Abbreviations: T1DM, type 1 diabetes mellitus; HCPs, healthcare professionals; USA, United States; UK, United Kingdom; SD, standard deviation; NR, not reported;

^a^ Sample consisted of young people with T1DM unless indicated otherwise

^b^ Number {a}/Number {b} in this column represents that the study met {a} criteria out of the total {b} criteria. For qualitative studies, we used the 10 criteria of CASP, and for mixed methods studies we used the 17 criteria from MMAT (see Appendix 3).
