# Supplementary material for: Experiences and Views of Young People and Health Care Professionals of Using Social Media to Self-Manage Type 1 Diabetes Mellitus: Thematic Synthesis of Qualitative Studies
Source: JMIR Pediatr Parent. 2024 May 29;7:e56919. doi: 10.2196/56919 (PMC11170052; doi:10.2196/56919)
Supplement: Multimedia Appendix 3 [file pediatrics_v7i1e56919_app3.docx]

**Table S1.** Critical appraisal skills programme (CASP) for assessment of qualitative studies^a^.

| Author  Publication year | Was there a clear  statement of the aims of  the research? | Is a qualitative  methodology  appropriate? | Was the research  design appropriate to  address the aims of the  research? | Was the recruitment  strategy appropriate to  the aims of the  research? | Was the data collected in  a way that addressed the  research issue? | Has the relationship  between researcher and  participants been  adequately considered? | Have ethical issues been  taken into consideration? | Was the data analysis  sufficiently rigorous? | Is there a clear statement  of findings? | How valuable is the  Research? | Criteria that met |
| --- | --- | --- | --- | --- | --- | --- | --- | --- | --- | --- | --- |
| Malik et al, [22]  2019 | Yes | Yes | Yes | Yes | Yes  In library setting or hospital conference room. | Yes  Endocrinologist, clinical staff and researchers conducted interviews | Yes  Debriefs after each focus group; Ethical approval granted. | Yes  Data saturation used | Yes | Yes | 10/10 |
| Nordfeldt et al, [44]  2013 | Yes | Yes | Yes | Yes  Strategic sampling used | Yes  Topic guide used, setting described. | Yes  Trained personnel conducted interviews | Yes  Ethic approval granted; Data storage discussed. | Yes  Iterative, resolved discussions, 2 independent researchers.  Comments: No mention of saturation | Yes | Yes  Further research explained | 10/10 |
| Clarke et al, [23]  2018 | Yes | Yes | Yes | Yes  Sampling to saturation | Yes  Sampling to saturation | Yes  Clinically trained staff conducted interviews | Yes  Ethical approval granted. | Yes  Followed guideline; Data saturation used.  Comments: Data analysis was conducted by one person. | Yes | Yes  Broad suggestions for health professionals and mental researchers regarding young patients with T1DM provided | 10/10 |
| Sawyer et al, [45]  2022 | Yes | Yes | Yes | Yes | Yes  Interview questions were informed by a survey. | Yes  Info about researchers given, and how interviews were structured | Yes  Ethical approval granted; Identifiable information removed | Can’t tell  Need further explanation of the data synthesis procedure; Did not mention how disagreements were resolved. | Yes | Yes | 9/10 |
| Chalmers et al, [39]  2022 | Yes | Yes | Yes | Yes  Sampling to saturation | Yes  Interview guide developed; Sampling to saturation | Yes  Trained researchers; followed Consolidated Criteria for Reporting Qualitative Research. | Yes  Ethical approval granted | Yes  Data saturation used; Biweekly debriefs to discuss interview summaries | Yes | Yes | 10/10 |
| Fergie et al, [40]  2016 | Yes | Yes | Yes | Yes  Recruited on and offline; Purposive sample (Did not explain what did this mean) | Yes ­­ | No | Yes  Ethical approval granted | Yes  Refinement agreed | Yes | Yes | 9/10 |
| Nordfeldt et al, [43]  2012 | Yes | Yes | Yes | Yes  Recruited through email invitation | Yes | Yes | Can’t tell | Yes  Iterative in-depth discussion sessions, a stepwise re-categorization and repeated validation | Yes | Yes | 9/10 |
| Yi-Frazier et al, [46]  2015 | Yes | Yes | Yes | Yes  From hospital and telephone or sent mail regarding the study | Yes | No | Yes  Protocol approved; Voluntary written informed consent obtained. | No  No mention of saturation or disagreements | Yes | Yes | 8/10 |
| Brady et al, [38]  2016 | Can’t tell  Not a clear statement about the aim | Yes | Yes | Yes  Recruited online and offline | Yes | No | Yes  Ethical approval granted; Identifiable information removed; Signed consent obtained. | No  Did not mention how many people analsyed, or how they resolved disagreement, and did not mention saturation. | Yes | Yes | 7/10 |

^a^The checklist for assessment of qualitative studies is based on the critical appraisal skills programme checklist [36]

**Table S2.** Mixed methods appraisal tool (MMAT) for assessment of mixed-methods studies^a^.

| Reference | Type of study component | Methodology quality criteria | Response  Yes/ No/ Unclear | Comments | Criteria that met |
| --- | --- | --- | --- | --- | --- |
| Ng et al, [42]  2019 | Screening questions (for all types) | Are there clear research questions  Does data collected allow these questions to be answered? | Yes  Yes | Could be clearer objectives | 16/17 |
|  | Qualitative | 1.1 Is the qualitative approach appropriate to answer the research question?  1.2 Are the qualitative data collection methods adequate to address the research question?  1.3 Are the findings adequately derived from the data?  1.4 Is the interpretation of results sufficiently substantiated by data? 1.5 Is there coherence between qualitative data sources, collection, analysis and interpretation? | Yes  Yes  Yes  Yes  Yes | Open ended questions easy when using questionnaire, didn’t discuss benefits of this method over any other type of data collection |  |
|  | Quantitative descriptive | 4.1 Is sampling strategy relevant to research question?  4.2 Is sample representative of population?  4.3 Are measurements appropriate?  4.4 Is the risk of nonresponse bias low?  4.5 Is the statistical analysis appropriate to answer the research question? | Yes  No  Yes  Yes  Yes | They mentioned in limitations the sample may only represent those who manage their T1D and who already use computer for info |  |
|  | Mixed methods | 5.1 Is there an adequate rationale for using a mixed methods design to address the research question?  5.2 Are the different components of the study effectively integrated to answer the research question?  5.3 Are the outputs of the integration of qualitative and quantitative components adequately interpreted?  5.4 Are divergences and inconsistencies between quantitative and qualitative results adequately addressed?  5.5 Do the different components of the study adhere to the quality criteria of each tradition of the methods involved? | Yes  Yes  Yes  Yes  Yes | Examples given where participants say opposing views |  |
| Malik et al, [41]  2021 | Screening questions (for all types) | Are there clear research questions  Does data collected allow these questions to be answered? | Yes  Yes |  | 15/17 |
|  | Qualitative | 1.1 Is the qualitative approach appropriate to answer the research question?  1.2 Are the qualitative data collection methods adequate to address the research question?  1.3 Are the findings adequately derived from the data?  1.4 Is the interpretation of results sufficiently substantiated by data? 1.5 Is there coherence between qualitative data sources, collection, analysis and interpretation? | Yes  Yes  Yes  Yes  Yes | Qualitative content analysis was used to identify themes |  |
|  | Quantitative descriptive | 4.1 Is sampling strategy relevant to research question?  4.2 Is sample representative of population?  4.3 Are measurements appropriate?  4.4 Is the risk of nonresponse bias low?  4.5 Is the statistical analysis appropriate to answer the research question? | Yes  No  Yes  Yes  Yes | They mentioned in limitations the small sample size due to the pilot nature of the study, which limits generalizability. |  |
|  | Mixed methods | 5.1 Is there an adequate rationale for using a mixed methods design to address the research question?  5.2 Are the different components of the study effectively integrated to answer the research question?  5.3 Are the outputs of the integration of qualitative and quantitative components adequately interpreted?  5.4 Are divergences and inconsistencies between quantitative and qualitative results adequately addressed?  5.5 Do the different components of the study adhere to the quality criteria of each tradition of the methods involved? | Yes  Yes  Yes  Unclear (no inconsistency was reported)  Yes |  |  |

^a^The checklist for assessment of mixed-method studies is based on the mixed methods appraisal tool [37]
