# Supplementary material for: Experiences and Views of Young People and Health Care Professionals of Using Social Media to Self-Manage Type 1 Diabetes Mellitus: Thematic Synthesis of Qualitative Studies
Source: JMIR Pediatr Parent. 2024 May 29;7:e56919. doi: 10.2196/56919 (PMC11170052; doi:10.2196/56919)
Supplement: Multimedia Appendix 4 [file pediatrics_v7i1e56919_app4.docx]

**Table S1.** Themes Identified in Each Study (numbers refer to the number of quotations coded under each theme).

| Analytic Themes | Descriptive themes | References | | | | | | | | | | |
| --- | --- | --- | --- | --- | --- | --- | --- | --- | --- | --- | --- | --- |
|  |  | [42] | [22] | [44] | [23] | [41] | [45] | [39] | [40] | [43] | [46] | [38] |
| **Differences in how young people interact with social media** | Passive versus active user  Engagement | 2 | 1 | 2 | 0 | 2 | 1 | 8 | 0 | 3 | 1 | 0 |
|  | Levels of T1DM disclosure | 0 | 0 | 12 | 0 | 0 | 0 | 8 | 0 | 0 | 0 | 0 |
| **Characteristics of social media platforms that influence their use and uptake for T1DM self-management** | Easier access to information and peer support | 0 | 6 | 0 | 0 | 0 | 0 | 0 | 0 | 1 | 0 | 0 |
|  | Platform design | 0 | 1 | 13 | 0 | 0 | 0 | 1 | 0 | 2 | 0 | 0 |
|  | Trustworthiness | 0 | 2 | 6 | 0 | 0 | 0 | 0 | 0 | 2 | 0 | 1 |
| **Social media as a source of information** | Catering for diverse information needs | 1 | 0 | 10 | 0 | 0 | 0 | 0 | 0 | 0 | 0 | 0 |
|  | Learning from peers’ experience | 2 | 2 | 1 | 0 | 1 | 0 | 3 | 0 | 3 | 0 | 0 |
|  | Educating others | 0 | 0 | 2 | 0 | 0 | 0 | 1 | 0 | 1 | 0 | 0 |
| **Impact on young people's coping and emotional wellbeing** | Emotional support and peer support | 2 | 3 | 0 | 4 | 6 | 2 | 8 | 5 | 0 | 1 | 0 |
|  | Humor and hope | 0 | 0 | 1 | 0 | 0 | 0 | 8 | 0 | 0 | 0 | 0 |
| **Impact on support from and relationship with HCPs and the healthcare service** | More direct support from HCPs | 0 | 5 | 0 | 0 | 0 | 0 | 0 | 0 | 2 | 0 | 0 |
|  | Better HCPs-patient relationships | 0 | 3 | 0 | 0 | 0 | 0 | 0 | 0 | 2 | 0 | 0 |
|  | Potential privacy concerns | 0 | 3 | 0 | 0 | 0 | 0 | 0 | 0 | 0 | 0 | 0 |
